# Ante-mortem diagnosis of unilateral pulmonary vein stenosis in a cat: a case report

**DOI:** 10.1186/s13028-025-00803-y

**Published:** 2025-04-23

**Authors:** Takuma Aoki, Takashi Miyamoto, Kota Kizaki, Asuka Ueshima, Kentaro Iwasaki, Takuya Kusaka, Haruko Terui

**Affiliations:** 1 Laboratory of Small Animal Surgery, School of Veterinary Medicine, Azabu University, 1-17-71 Fuchinobe, Chuo-ku, Sagamihara, Kanagawa 252-5201 Japan; 2 Azabu University Veterinary Teaching Hospital, Azabu University, 1-17-71 Fuchinobe, Chuo-ku, Sagamihara, Kanagawa 252-5201 Japan; 3 Kodama Kyodo Hospital, 2-5-21 Kyodo, Setagaya-ku, Tokyo 156-0052 Japan; 4 Ogikubo Momoi Animal Hospital, 2-2-3 Momoi, Suginami-ku, Tokyo 167-0034 Japan

**Keywords:** Case report, Computed tomography, Echocardiography, Pulmonary hypertension, Pulmonary oedema

## Abstract

**Background:**

Pulmonary hypertension (PH) detection in cats may be challenging. Pulmonary venous stenosis (PVS) is rare in cats and can lead to PH. The only reported PVS case received a post-mortem diagnosis. Imaging during the cat's lifetime established the diagnosis in this case.

**Case presentation:**

A 2 year-old Norwegian Forest cat was diagnosed with pulmonary oedema and PH secondary to cor triatriatum sinister (CTS) and showed improved breathing following two subcutaneous furosemide treatments, 1 and 2 mg/kg, during an overnight stay at the referral veterinary hospital. Sildenafil alone (0.69 mg/kg, PO, BID) was prescribed post-discharge to address PH without diuretics. Post-discharge from the referral veterinary hospital, collapse and pre-syncope were suspected to be due to PH. Consequently, sildenafil was titrated weekly, starting at 1.09 mg/kg BID and increasing to 1.63 mg/kg BID. Pre-syncope and collapse resolved, and pulmonary opacities reduced considerably, although concerns remained that increased pulmonary blood flow to suspected CTS from sildenafil might worsen cardiogenic pulmonary oedema. The patient was also treated with rivaroxaban (2.5 mg/head, SID), considering the increased risk of thrombus formation due to blood flow stasis and endothelial damage. Thirty-eight days later, the cat presented for the first time to our hosipital (Azabu University Veterinary Teaching Hospital) for examination. On echocardiography, a continuous mosaic blood flow (maximum and minimum velocity, 3.14 m/s; estimated pressure gradient, 39.4 mmHg) was observed in two enlarged pulmonary veins. Pulmonary artery enlargement (main pulmonary artery to thoracic aorta ratio: 1.90), pulmonary vein stenosis (PVS), and diffuse bilateral ground-glass lung opacities were observed using computed tomography. PH with unilateral PVS involving two out of the three right pulmonary veins, specifically the right cranial and right middle pulmonary veins, along with pulmonary parenchymal disease, was diagnosed. The cat was further treated with furosemide (1 mg/kg, BID, PO) with no clinical signs but succumbed to acute dyspnoea 51 days after the first visit.

**Conclusions:**

Unilateral PVS should be considered in young cats with a localised alveolar pattern and no left atrial enlargement, because the prognosis may be poor. Severe PH with PVS may coexist with lung disease. If sildenafil is used, it should be started at a low dose and monitored closely.

## Background

Pulmonary hypertension (PH) in cats is challenging to detect as it lacks obvious clinical signs, especially before the condition becomes severe [1, 2]. Diagnostic options for pulmonary hypertension in cats are limited owing to challenges associated with invasive techniques such as right heart catheterization. Measuring pulmonary artery pressure via catheterization is not practical in cats because of technical challenges, high risks to the patient, and the invasive nature of the procedure. Unlike that in dogs, the estimation of pulmonary artery pressure using echocardiography with tricuspid regurgitation or pulmonary regurgitation has not been thoroughly investigated in cats. Furthermore, echocardiography, although being a non-invasive alternative, has limitations in accuracy and operator dependency. Additionally, factors such as limited awareness among veterinarians and the cost or availability of diagnostic tools further exacerbate the issue. These constraints hinder definitive diagnosis and potentially contribute to the underestimation of pulmonary hypertension prevalence in feline populations. Pulmonary venous stenosis (PVS) is rare in cats and can lead to severe post-capillary PH without left atrial enlargement due to increased pulmonary vein pressure [3]. Only one previous study has reported a case of PVS and resultant PH in cats, with this diagnosis obtained post-mortem [3]. Feline PH is classified into six groups based on underlying aetiologies, similar to canine PH. Although the treatment of the underlying disease is prioritized, sildenafil, a phosphodiesterase type 5 (PDE5) inhibitor, is commonly used across many groups to achieve pulmonary vasodilation when pulmonary arterial pressure is elevated [2]. Sildenafil enhances the activity of endogenous nitric oxide by increasing cyclic guanosine monophosphate, thereby reducing pulmonary vascular constriction and lowering pulmonary arterial pressure [1, 2]. However, in post-capillary PH associated with left-sided heart disease, increased pulmonary blood flow with sildenafil may elevate the risk of cardiogenic pulmonary oedema [1]. Conversely, in cases of PH secondary to pulmonary fibrosis in cats, clinical signs associated with PH have been reported to improve [2]. Here, we report a case of feline suspected PH secondary to congenital PVS and parenchymal lung disease, detected using echocardiography and computed tomography (CT) scanning, in which sildenafil was administered.

## Case presentation

**Fig. 1:**
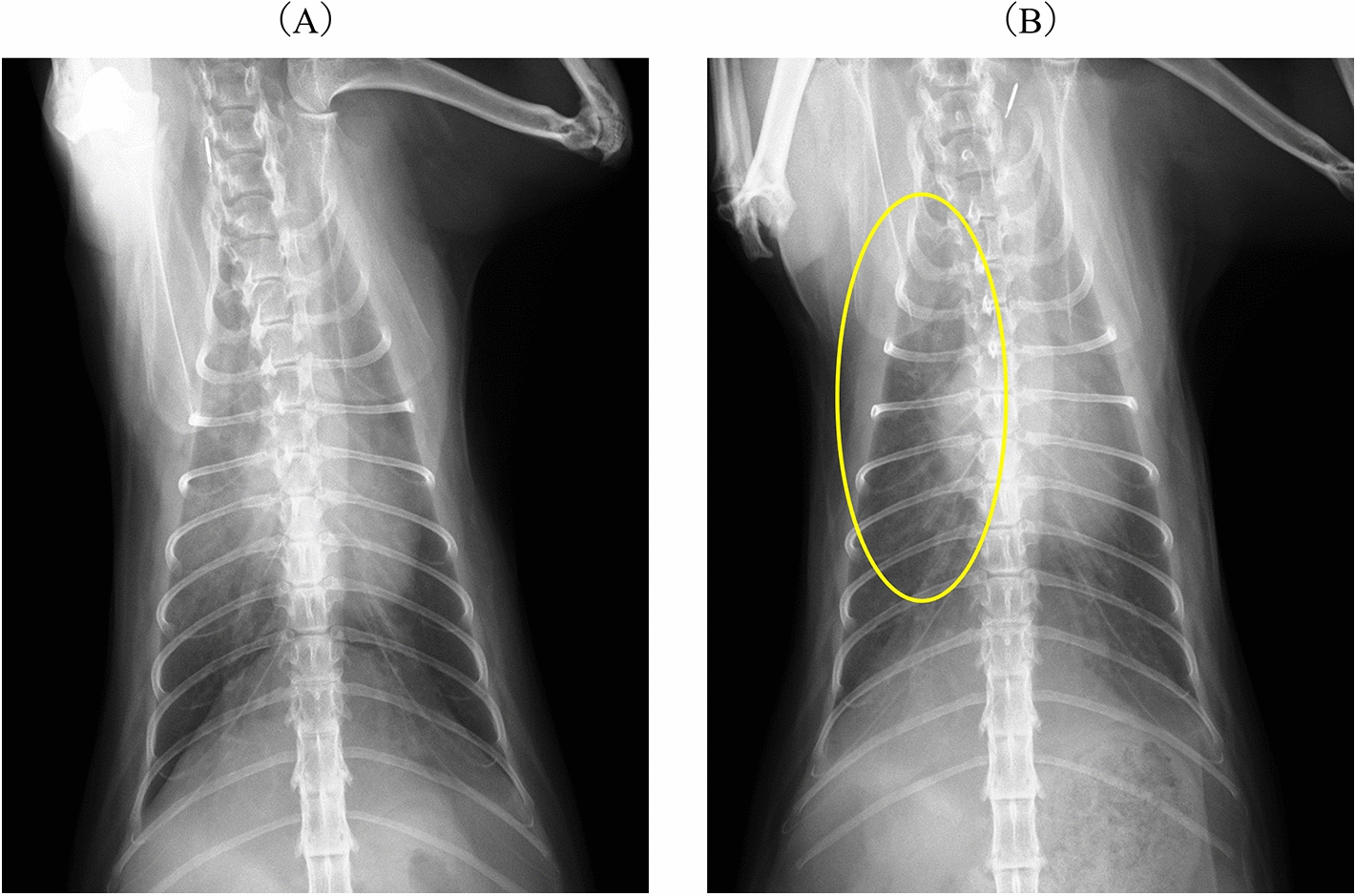
Thoracic radiography findings (dorsoventral view). Images obtained by the referring veterinarian. At initial presentation (**a**), an alveolar pattern is observed in the right cranial lobe. **b** After 19 days of sildenafil administration

**Fig. 2:**
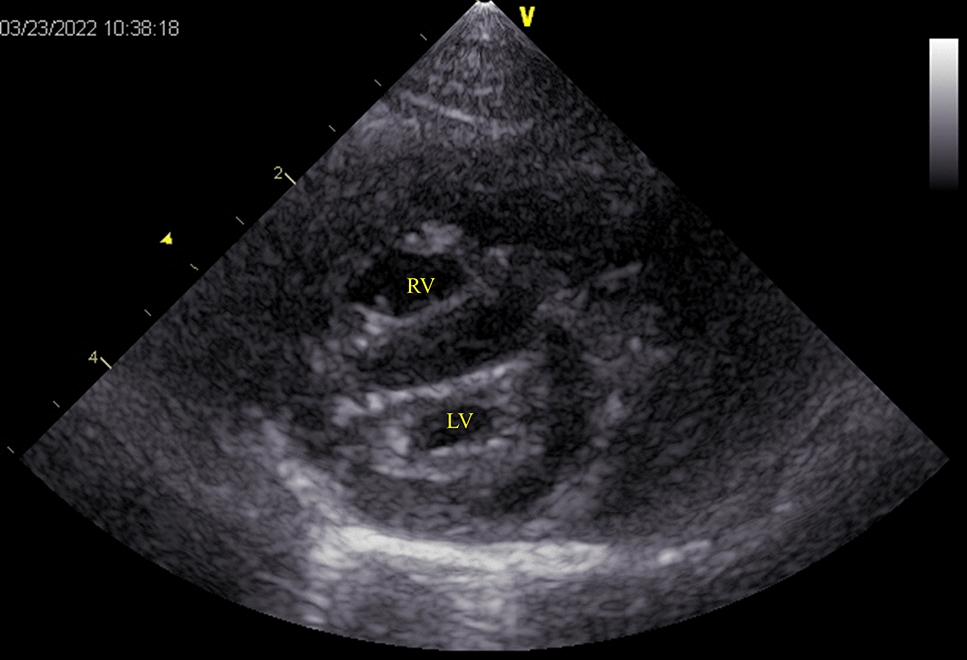
Echocardiography findings. A right parasternal short-axis view at the level of the left ventricle revealed systolic flattening of the interventricular septum and compression of the left ventricle. Please refer to the echocardiographic video (Supplementary Video 1). LV: left ventricle; RV: right ventricle

**Fig. 3:**
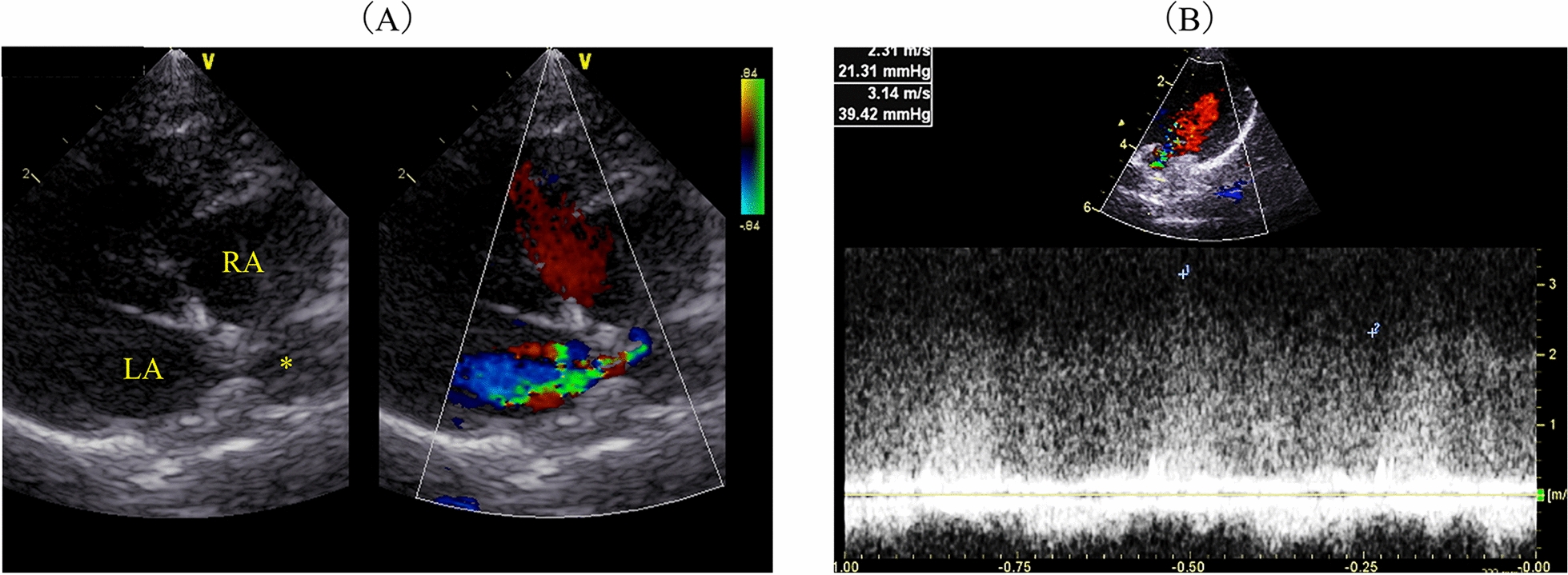
Echocardiographic findings. **a** Simultaneous transthoracic echocardiography and colour-flow Doppler imaging of the left atrium. The colour-flow Doppler imaging reveals turbulent flow from the dilated pulmonary vein (*) into the left atrium, which is also continuous. **b** Continuous Doppler echocardiography shows continuous blood flow from the pulmonary vein, with a peak velocity of 3.14 m/s through the pulmonary vein stenosis, indicating severe pathology. LA: left atrium, RA: right ventricle

**Fig. 4:**
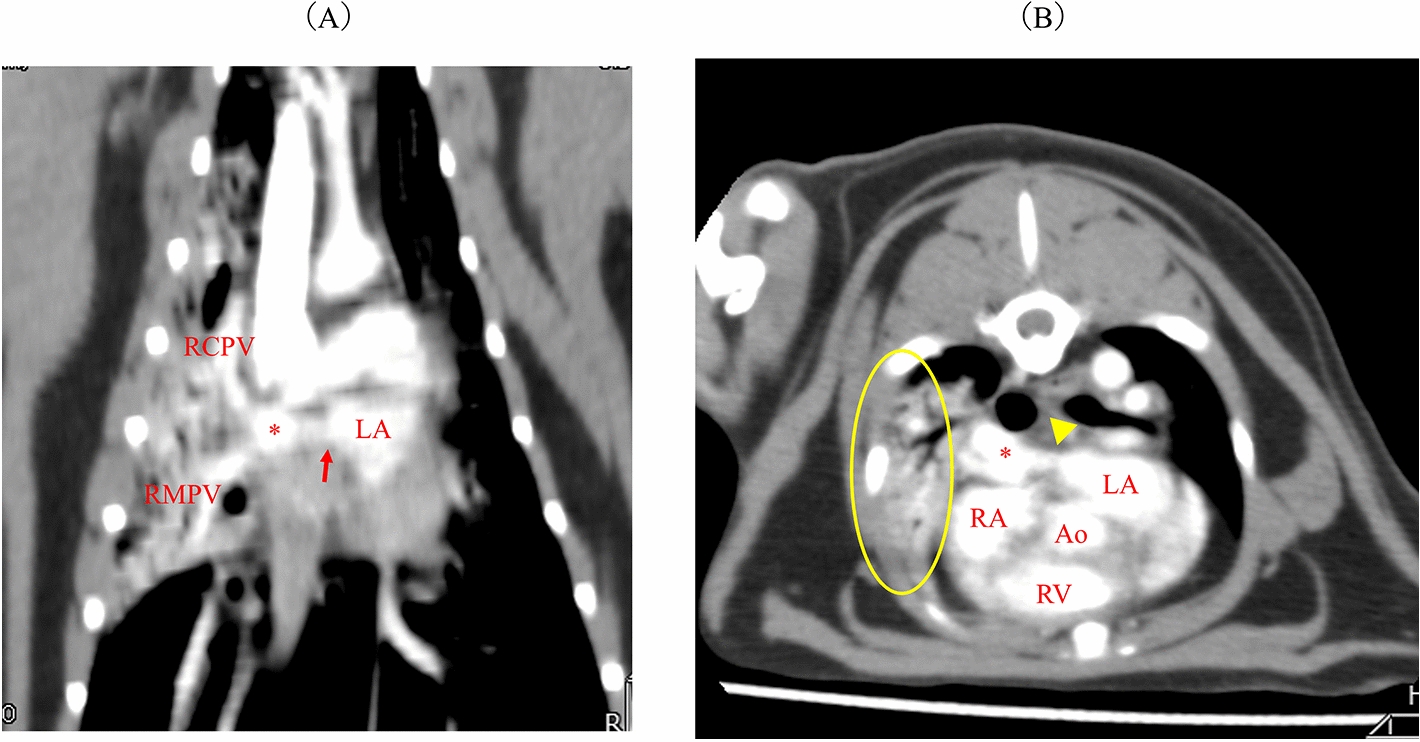
Findings from computed tomography scans. **a** A coronal section showing stenosis between the right cranial pulmonary vein (RCPV) and right middle pulmonary vein (RMPV), forming an enlarged short common trunk (*), with a stenotic ostium leading to the left atrium (LA; red arrows). **b** An axial section illustrating the stenosis (yellow arrowhead) between the enlarged short common trunk (*) and the LA. Consolidation and air bronchograms are evident in the right middle lobe (yellow ellipses). Ao, aorta; RA, right atrium; RV, right ventricle

**Fig. 5:**
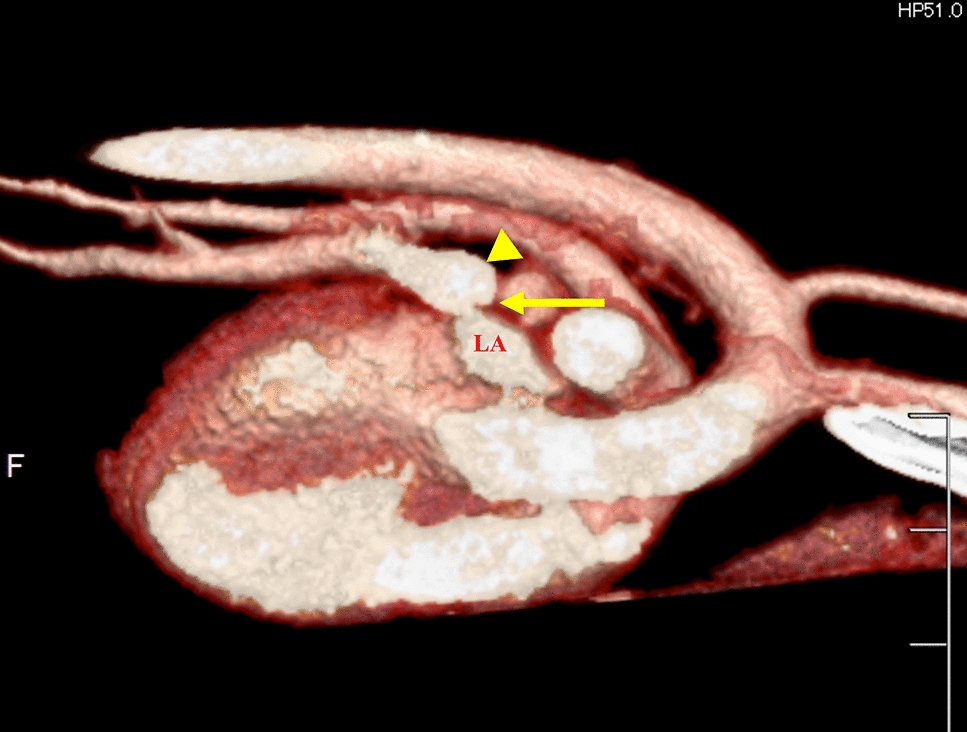
Findings from computed tomography (CT) scans. A right-side view of a contrast-enhanced three-dimensional CT scan demonstrates a stenotic ostium (yellow arrow) in the enlarged short common tract (yellow arrowhead) formed by the right cranial and middle lobe veins (not visible on the image), connecting to the left atrium (LA)

**Fig. 6:**
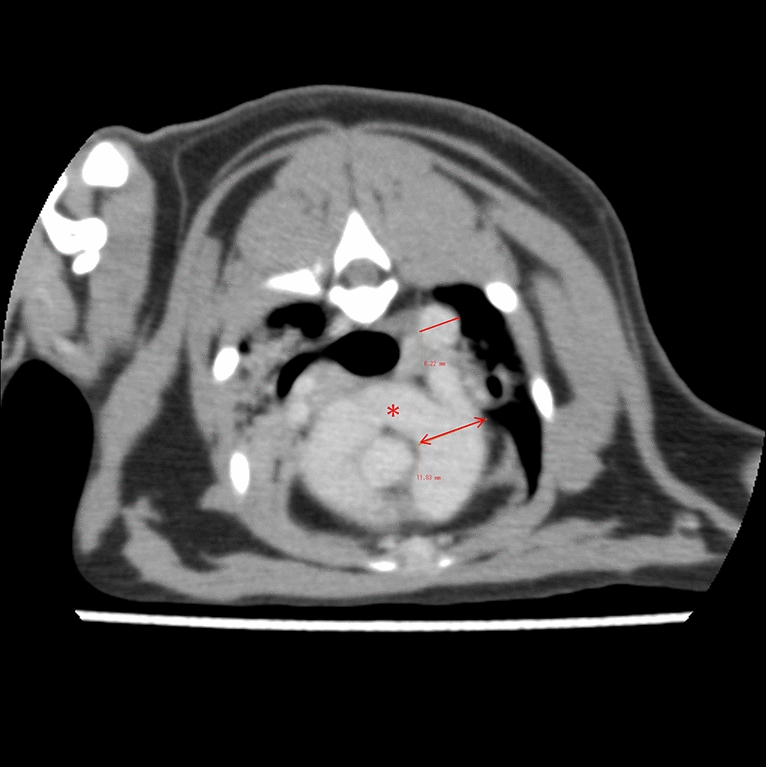
Findings of computed tomography scanning. In the axial view, the diameter of the pulmonary artery (double arrows) is markedly increased relative to that of the descending aorta (solid line). In addition, significant dilation of the right pulmonary artery is noted (*)

**Fig. 7:**
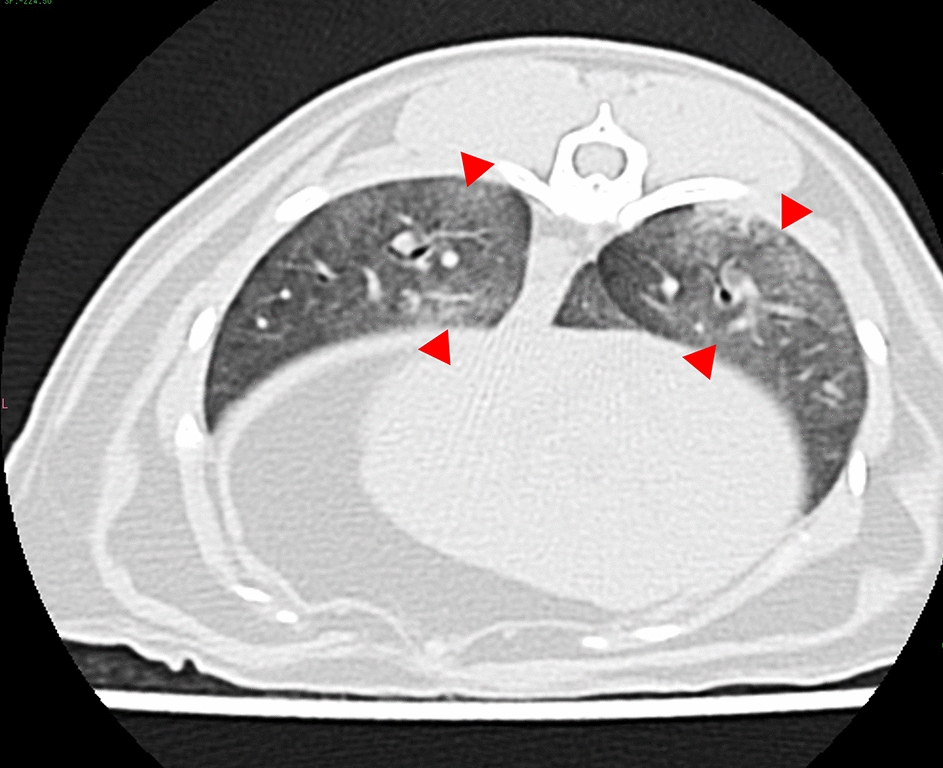
Findings of computed tomography scanning. An axial view of the thoracic posterior lobes shows diffuse ground-glass opacities (red arrowheads)

A 2 year-old, spayed, female Norwegian Forest cat (4.94 kg, body condition score: 4/9) presented to referral veterinarian with anorexia and dyspnoea. The sleeping respiratory rate was not assessed at home in this case. Therefore, its potential utility in monitoring respiratory status could not be evaluated. The cat was diagnosed with PH associated with pulmonary oedema secondary to suspected cor triatriatum sinister (CTS), with the diagnosis supported by the presence of an obstructive membrane at the basal portion of the left atrial appendage [4] and ventricular septal systolic flattening. On day 1, 2 mg/kg furosemide was administered subcutaneously twice, and the patient was hospitalised overnight in a 40% oxygen chamber (ICU-MENIOS compact; Tokyo Menix Co., Ltd., Tokyo, Japan). The following day, after the patient spent more than 5 min in room air, arterial blood gas analysis revealed oxygen and carbon dioxide partial pressures of 62.9 mmHg (reference range: 102.9 ± 15 mmHg [5]) and 36.9 mmHg (reference range: 33.6 ± 7 mmHg), respectively. The patient was discharged and treated with sildenafil (0.69 mg/kg, BID, PO) alone. The referring veterinarian administered sildenafil based on a diagnosis of hypoxemia and non-cardiogenic pulmonary oedema secondary to pulmonary hypertension. This diagnosis was made on the basis of persistent pulmonary opacities observed on thoracic radiographs despite furosemide therapy. After discharge, the patient experienced occasional collapse and pre-syncope; however, during a follow-up visit 1 week later, improvements in breathing and appetite were noted. Notably, an oxygen concentrator and chamber were rented to manage hypoxemia. However, the cat did not utilize the chamber, possibly owing to the stabilization of its respiratory condition. At 1 week post-discharge, thoracic radiography revealed residual pulmonary opacities. Given the improvement in respiratory pattern and appetite, the referring veterinarian considered sildenafil to be effective and decided to increase the dose to 1.09 mg/kg BID to further address pulmonary opacities. After an additional week, the patient’s respiratory status had normalised, and activity levels improved with no further episodes of pre-syncope. The sildenafil dose was increased to 1.63 mg/kg BID because of residual abnormal pulmonary opacities in the right cranial lung field on the radiography after an additional week. Subsequently, pre-syncope or collapse was not observed, and pulmonary opacities reduced markedly on thoracic radiography 19 days after sildenafil administration (Fig. 1), although, as previously mentioned, sildenafil carries a high risk of exacerbating pulmonary oedema caused by CTS. The patient was additionally treated with rivaroxaban at a fixed dose of 2.5 mg once dayily per cat, prescribed by the referring veterinarian to address the potential risk of thrombosis due to blood flow stagnation associated with CTS. Thirty-eight days after the initial clinical manifestations, the cat was referred to the Department of Cardiology and Respiratory Medicine at our animal hospital for a detailed examination. At the time of presentation to our facility, its health status was improving. The patient’s vital signs were recorded (body temperature, 38.1 °C; respiratory rate, 78 breaths/min; heart rate, 180 beats/min; and no heart or secondary murmurs). The average systolic blood pressure assessed using the Doppler method was 124 mmHg. Thoracic radiography revealed an alveolar pattern in the anterior lobe, a vertebral heart scale of 8.2, and mild cardiac enlargement. Transthoracic echocardiography was performed using an ultrasound unit (Vivid E9; GE Healthcare Co., Ltd., Tokyo, Japan) with 6- to 12-MHz phased-array transducers. Transthoracic echocardiography revealed right cardiac enlargement, pulmonary artery dilatation, ventricular septal systolic flattening, and an increased pulmonary artery to aortic ratio (MPA/Ao: 1.36; normal range: < 1.0) [1, 2], indicating PH (Fig. 2; Supplementary Video 1). In the Doppler method, the blood flow velocity in the right ventricular outflow tract was 1.03 m/s, with no tricuspid regurgitation, pulmonary valve regurgitation, or intracardiac shunt observed. No obstructive membrane was observed in the left atrium; accordingly, cor triatriatum sinister was ruled out. The LA/Ao was 1.26, and the left ventricular end-diastolic diameter was 1.45 cm. LV fractional shortening was 55.64%. The right pulmonary vein, which had a stenotic ostium at the left atrium, showed continuous and increased blood flow velocities (minimum and maximum velocities were 2.42 m/s and 3.14 m/s, respectively, with estimated pressure gradients of 21.31 mmHg and 39.42 mmHg; Fig. 3). The client was informed about the anaesthetic procedure and CT imaging for the purpose of making a definitive diagnosis through the process of informed consent, and their agreement was obtained prior to proceeding. The procedure involved CT imaging (Aquilion Prime SP; Canon Medical Systems Corporation, Tochigi, Japan) conducted under general anaesthesia. A non-ionic contrast agent (Iohexol 300 mg; dose, mL/kg) was administered intravenously, and images were obtained over a duration of 15 s. Triphasic angiography was used for the evaluation of pulmonary and cardiac structures. CT scans revealed a short common trunk formed by the convergence of the right cranial and right middle pulmonary veins. A stenotic ostium was identified between this trunk and the left atrium (Figs. 4 and 5). Additional findings included pulmonary artery enlargement, with a main pulmonary artery-to-thoracic aorta ratio of 1.90 (Fig. 6; normal range: < 1.0) [2, 6], and consolidation in the right cranial and middle pulmonary lobes (Fig. 4). Moreover, ground-glass opacities (− 300 to − 100 Hounsfield units [HU], Fig. 7) were observed in the lung parenchyma (− 500 and − 600 HU), pulmonary vessels (approximately 150 HU), and bronchial walls (approximately 100 HU). Consequently, segmental PH due to partial PVS was diagnosed. We also diagnosed the cat with a possible pulmonary parenchymal disease in the cat based on CT findings, which, along with partial PVS in the right cranial and middle lung lobes, may have contributed to PH.

When informed of the poor prognosis, the owner declined a lung biopsy. Because we suspected pulmonary oedema from CT findings, we administered 1 mg/kg intravenous furosemide (Lasix 10 mg injection; Sanofi KK, Tokyo, Japan) on the day of presentation and prescribed 1 mg/kg BID oral furosemide (Lasix 10 mg tablet; Sanofi KK, Tokyo, Japan) starting the following day. We also requested the referring doctor to prescribe sildenafil and rivaroxaban. Sildenafil is likely to exacerbate pulmonary oedema caused by pulmonary vein stenosis. In this case, the initial clinical course appeared to show a therapeutic response to sildenafil alone, leading to its continued use alongside the addition of furosemide. However, at the time of presentation to our hospital, the patient was experiencing cardiogenic pulmonary oedema. Notably, upon discharge, after the patient was managed with furosemide in a 40% oxygen chamber (ICU-MENIOS; Tokyo Menix Co., Ltd., Tokyo, Japan) for one night, the respiratory rate was 48 breaths/min, suggesting that the tachypnoea observed at the time of presentation was not only due to stress but was also significantly associated with pulmonary oedema. The patient visited our hospital only once, and its condition appeared stable with no episodes of syncope or dyspnoea reported thereafter. However, the patient experienced acute dyspnoea and presumably succumbed to pulmonary oedema 51 days after the last hospital visit. A necropsy could not be performed as the owner did not provide consent, which precluded determination of the definitive cause of death, the presence of pulmonary lesions, and their potential diagnosis.

## Discussion and conclusion

PVS is a rare cardiovascular disease; to date, only one veterinary case report of a Maine Coon cat with congenital PVS confirmed using echocardiography and necropsy after ethical euthanasia has been reported [3]. PVS is a rare congenital or acquired cardiovascular disease, even in humans [7]. Although it depends on the number of pulmonary veins with stenosis and the degree of stenosis, PVS-induced pulmonary oedema and PH contribute to high morbidity and mortality in infants and young children [8, 9]. PVS is primarily recognized as a congenital anomaly and is often associated with conditions such as total anomalous pulmonary venous return, where abnormal pulmonary vein connections are present. PVS can also develop postoperatively following the surgical correction of total or partial anomalous pulmonary venous return, particularly in infants and children [10]. Recently, PVS has also been linked to parenchymal lung diseases, such as bronchopulmonary dysplasia, which frequently occur in premature infants (< 37 weeks) [7, 11]. Furthermore, reports have described PVS as a complication following atrial fibrillation ablation procedures [8, 12]. Congenital PVS is typically characterized by stenosis at the pulmonary vein ostia, whereas cor triatriatum sinister (CTS) involves an obstructive membrane within the left atrial cavity [3]. In this case, transthoracic echocardiography ruled out CTS by confirming the absence of an obstructive membrane in the left atrium. Furthermore, the presence of a continuous blood flow waveform through a stenotic ostium in the pulmonary vein raised suspicion of PVS. However, in cases where the pulmonary veins cannot be adequately visualised, the high spatial resolution of CT imaging enables accurate diagnosis [8]. In this case, CT imaging confirmed the absence of an obstructive membrane in the left atrium and identified a stenotic ostium in the pulmonary veins, leading to a definitive diagnosis of PVS.

The PVS in the present case was likely congenital, as the patient was relatively young. In a young cat with severe PH due to congenital PVS in a previous report, an autopsy revealed stenosis in all pulmonary veins except the left cranial pulmonary vein [3]. Cats have been reported to have two types of right cranial and middle pulmonary vein ostia: those that join at the common trunk and those that independently flow into the pulmonary venous drainage system [13]. In the present case, the right cranial and middle pulmonary veins formed a short common trunk, which had a stenotic ostium at its junction with the left atrium. Pulmonary venous flow patterns typically consist of a systolic S wave and a diastolic D wave, both returning to baseline. PVS is suspected when either the S or D wave exceeds 0.7 m/s [14]. Additionally, the mean pressure gradient can be calculated from the two flow waveforms, and a value of 3 mmHg or more suggests PVS. In the present case, the calculation of an accurate mean pressure gradient was particularly challenging owing to the absence of an ECG, which made it difficult to synchronize measurements, and the unclear edges of the Doppler signal caused by the continuous blood flow waveform. However, the continuous flow pattern itself supported the diagnosis of PVS, and the high pulmonary venous pressure could also be inferred from the peak flow velocity. The pressure gradient between the pulmonary vein and the left atrium, measured by cardiac catheterization, is considered indicative of severe pulmonary vein stenosis when it is 10 mmHg or more [14]. Unlike catheterization, Doppler-derived pressure gradients are less accurate and can be influenced by factors such as blood flow and measurement conditions. The maximum blood flow velocity in the stenotic ostium in the present case was 3.14 m/s (gradient pressure [PG] 39.4 mmHg), which is diagnostic for PVS in humans with a blood flow velocity of 1.1–1.5 m/s [15, 16]. As the prior case report of a cat with PVS revealed severe PVS with a blood flow velocity of 2.6 m/s (PG 27.0 mmHg) [3], the present case likely had severe PH in the affected lungs. However, unlike in the previous report, this case only had stenosis of the right cranial and middle pulmonary veins, which may have resulted in a relatively long survival. Furthermore, the cat had complete flattening of the ventricular septum during systole, indicating the possibility of PH [17]. However, the association of PH with the involvement of just two pulmonary vein stenoses remains questionable. In humans, segmental PH is described as pulmonary vascular remodelling and pulmonary hypertension in some, but not all, lung lobes and pulmonary segments [18]. For segmental PH to severely increase right ventricular pressure, multiple pulmonary lobes would need to be affected. In patients with PVS complicated by Maze surgery for atrial fibrillation, complete occlusion of the left superior PV and 30–40% stenosis of the inferior PV resulted in a moderate increase in right ventricular pressure [16]. Even in pigs with experimental treatment affecting over half of the lungs, the mean pulmonary artery pressure was 34 ± 9 mmHg [19]. Severe PH was suspected based on the presence of septal flattening [17, 20]; unfortunately, no clear evidence of PH severity was obtained due to the lack of a Doppler- and/or catheter-derived estimate of PA pressure. In this case, CT scans revealed no significant abnormalities or evidence of pulmonary vein hypertension in the pulmonary veins of the lung lobes, except for the narrowed short common trunk connecting the right cranial and right middle pulmonary veins. Therefore, although ground-glass opacities can be associated with various conditions such as pulmonary oedema and tumours, in this instance, they were more likely to indicate parenchymal lung disease. PVS can be associated with bronchopulmonary dysplasia [7, 11], and the current case of PH could be related to partial PVS and bronchopulmonary dysplasia. However, of the various features of bronchopulmonary dysplasia, only emphysema was observed in the CT findings of the present case. Other features, including bronchial wall thickening and subpleural opacity, were not observed [21, 22]. Accordingly, we concluded that bronchopulmonary dysplasia was unlikely. Consequently, the patient was diagnosed with parenchymal lung disease, a condition clearly present in this case, although histopathological examination would be required for a definitive diagnosis.

Pulmonary venous-occlusive disease (PVOD)/pulmonary capillary haemangiomatosis (PCH) involves smaller pulmonary vein lesions than PVS and presents with postcapillary pulmonary hypertension [23]. PVOD/PCH causes progressive dyspnoea, and pulmonary vasodilators can exacerbate pulmonary oedema owing to increased blood flow [24]. Although sildenafil and pimobendan were ineffective in managing dyspnoea associated with PVOD in dogs, they did not worsen pulmonary oedema [25]. Conversely, a cat with PCH was reported to develop pulmonary oedema within 45 min of sildenafil administration (0.9 mg/kg, PO) and was euthanized [26]. Diagnostic features of PVOD/PCH on CT include perivascular nodular ground-glass opacities, fissure lines, and enlargement of lobar pulmonary arteries [23]. Importantly, these diagnostic criteria were not met in our case.

In cats, similar to dogs, PH may be classified into six groups: Group 1, pulmonary arterial hypertension; Group 2, PH due to left heart disease; Group 3, PH due to respiratory disease or hypoxaemia; Group 4, PH due to thrombotic or embolic disease; Group 5, PH due to parasites; and Group 6, PH due to unclear or multifactorial mechanisms [1, 2]. In this case, PH was due to causes from Groups 2 and 3, and its classification was Group 6. A previous study reported that in Group 2 cases of PH, defined by a tricuspid regurgitation velocity > 2.7 m/s, PH was observed in 17% of cats with congestive left heart failure [27]. In contrast, in dogs with mitral valve insufficiency, PH was diagnosed using a tricuspid regurgitation velocity ≥ 3 m/s, with 52.7% of dogs in Stage C (those with current or past congestive heart failure) showing evidence of PH. In Stage B2, characterised by cardiac enlargement without signs of congestion, the prevalence of PH was considerably lower at 24.0% [28]. Among cats with congestive left heart failure, the prevalence of PH associated with acquired myocardial disease ranged from 13 to 37.5%. Notably, in cases of congenital heart disease (CHD), the prevalence reached 100%, although details about the specific types of CHD were not provided [27]. Additionally, PH was more frequently observed in cases of chronic congestive heart failure compared to acute congestive heart failure [27]. In the present case, the cat may have developed PH due to congenital heart disease, PVS, and chronic congestive heart failure at the age of 2 years. PH in Group 3 cats primarily results from alveolar hypoxia and pulmonary artery constriction caused by chronic lung disease and hypoxaemia, with the feline pulmonary vasculature being highly sensitive to effects of hypoxia and chronic respiratory disease, likely contributing to PH development [29]. The progression of primary pulmonary parenchymal disease, such as pulmonary fibrosis, in cats can lead to the development of PH through the accumulation of lung tissue damage and associated collagen deposition, which impairs respiratory function and causes hypoxia [30]. In this case, the arterial partial pressure of oxygen was 62.9 mmHg, indicating hypoxaemia. However, pinpointing the exact cause of hypoxaemia remains challenging, because it is likely multifactorial.

Using pulmonary vasodilators is not recommended for humans in Group 3, as they increase blood flow to poorly ventilated areas, thereby worsening the ventilation-blood flow imbalance [31]. However, phosphodiesterase-5 inhibitors have improved the quality of life and survival in dogs with PH secondary to obstructive and restrictive lung disease, although administration should be considered with caution [32, 33]. A previous report revealed that sildenafil was effective in cats with pulmonary fibrosis [2]. Furthermore, in the treatment of Group 2 PH, therapies aimed at reducing left atrial pressure should be implemented. Sildenafil may increase pulmonary venous return, potentially exacerbating pulmonary venous hypertension [1, 2]. In this case, before the cat visited our clinic, it received a diuretic only on the first day of respiratory distress; subsequently, it received sildenafil, which appeared to elicit a dose-dependent response and resulted in the disappearance of syncope and abnormal opacities in the lungs. However, after the treatment with sildenafil alone, oedema secondary to pulmonary venous hypertension was strongly suspected. The presence of pulmonary oedema and PH limited to the right cranial and right middle lobes likely contributed to the relatively stable respiratory condition. Conversely, pulmonary arterial pressure was not estimated before or after sildenafil administration in this case; sildenafil may have shifted blood flow to pulmonary vessels other than the right cranial and middle lobes, resulting in reduced blood flow in the right anterior and middle lobes and improved congestion. The efficacy of sildenafil in this case, where the cat was suspected to have Group 6 PH secondary to left heart and lung parenchymal diseases, cannot be confirmed. However, the findings suggest that it did not immediately worsen the pulmonary oedema in the lungs affected by PVS. To the best of our knowledge, no reports exist on the pharmacokinetics of sildenafil in cats. Regarding the dosage, the cat with PCH in the previously mentioned case report [26] received a single oral dose of 0.9 mg/kg. That cat was euthanized after developing congestive left heart failure; however, it could not be determined whether the dosage of sildenafil caused pulmonary vasodilation leading to congestive left heart failure. In another report of a cat with Eisenmenger syndrome due to an atrial septal defect that exhibited cyanosis and open-mouth breathing, sildenafil was initially administered at 0.25 mg/kg BID, and as the disease progressed, the dosage was gradually increased to 1.6 mg/kg BID because of syncope. The clinical symptoms ameliorated, and sildenafil was administered for a total of 10 months without any obvious side effects [34]. Another report of three cats with bidirectional or right-to-left shunting patent ductus arteriosus documented that sildenafil was used at higher doses, ranging from 1.8 to 5.1 mg/kg [35]. The criteria for administering sildenafil to cats appear to be respiratory distress and syncope related to PH, although the dosage has not been adequately studied [2]. However, because sildenafil may exacerbate congestive left heart failure depending on the PH condition, particularly in cases of vascular obstruction, it is necessary to start with a low dose and carefully monitor the patient in the veterinary hospital for over 1 h. This recommendation is based on a previous case report [26] in which a cat with PCH developed pulmonary oedema within 45 min of sildenafil administration. In the present case, the final dose of sildenafil was 1.63 mg/kg BID. However, because the underlying disease in this case differed from those in the previous reports and changes in pulmonary artery pressure before and after sildenafil administration could not be measured, it remains unclear whether this dosage of sildenafil had a significant effect in achieving pulmonary vasodilation.

Our interpretation of the diagnosis and treatment in this case has limitations. Sildenafil treatment was initiated by the referring veterinarian from the beginning, and diuretics were also administered, preventing determination of sildenafil’s true effect. In our veterinary hospital, we did not perform arterial blood gas analysis, which prevented us from clarifying the relationship between PH and hypoxaemia, as hypoxaemia was not confirmed at our facility. Furthermore, because the cat’s owner resided far away, the cat was not treated solely at our hospital, and we could not verify the changes in pulmonary arterial pressure before and after treatment or during the chronic course of the disease. Additionlly, although echocardiography and CT scan findings confirmed the diagnosis of PVS, a definitive determination of the cause of death and the exact nature of the pulmonary lesions could not be established, as an autopsy was not performed. PH was suspected because of systolic flattening of the interventricular septum on echocardiography and significant dilation of the pulmonary artery on CT and echocardiography findings; however, the presence and severity of PH remained unclear due to the absence of tricuspid regurgitation. In this case, the administration of sildenafil did not immediately exacerbate congestive left heart failure, unlike that in previous reports. However, the cat ultimately died of pulmonary oedema. It remains unclear whether the pulmonary oedema was due to the progression of PVS or a long-term increase in pulmonary venous return resulting from pulmonary vasodilation caused by sildenafil. The long-term effects of sildenafil administration on such conditions need to be observed at a single facility over an extended period. Interventional approaches for CTS, such as percutaneous transseptal balloon dilatation, have been reported in cats [36]. However, these techniques are specific to CTS and not directly applicable to the present case involving PVS. Although no established treatment currently exists for PVS in cats, various interventional techniques have been reported in human medicine. For example, percutaneous high-pressure balloon dilation has shown some efficacy, and stent placement reportedly offers superior long-term outcomes [8]. These approaches may offer future therapeutic potential for veterinary patients, although their clinical applicability in cats remains uncertain.

In conclusion, unilateral PVS should be considered as a differential diagnosis in young cats with an alveolar pattern localised to specific lung lobes. The diagnosis can be made using echocardiography and CT; however, the prognosis remains poor. When severe PH is observed to a degree that cannot be explained by unilateral PVS alone, the possibility of concurrent parenchymal lung disease should be considered. In such cases, sildenafil is expected to worsen pulmonary oedema in the affected lungs, although it did not immediately exacerbate the condition in the present case. The use of sildenafil in cats with respiratory distress or syncope due to PH should be considered; however, treatment should start with a low dose, and the patient must be carefully monitored even after administration.

## Data Availability

The datasets used and/or analysed during the current study are available from the corresponding author on reasonable request.

## Abbreviations

ACVIM: American College of Veterinary Internal Medicine
BID: Bis in die
CHD: Congenital heart disease
CT: Computed tomography
HU: Hounsfield units
LA: Left atrium
LV: Left ventricle
MPA/Ao: Main pulmonary artery to thoracic aorta ratio
PH: Pulmonary hypertension
PO: Per os
PVOD/PCH: Pulmonary veno-occlusive disease/pulmonary capillary haemangiomatosis
PVS: Pulmonary venous stenosis
RCPV: Right cranial pulmonary vein
RMPV: Right middle pulmonary vein
RV: Right ventricle
SID: Semel in die
TDI: Tissue doppler imaging
